# How is individualized nursing care documented in nursing records of cancer patients: A qualitative content analysis

**DOI:** 10.1186/s12912-025-03230-6

**Published:** 2025-05-22

**Authors:** Ingerd Irgens Hynnekleiv, Tove Giske, Kristin Heggdal

**Affiliations:** 1 Centre of Diakonia and Professional Practice, VID Specialized University, Oslo, 0310 Norway; 2 Faculty of Health Sciences, VID Specialized University, Bergen, Norway; 3 Faculty of Health Sciences, VID Specialized University, Oslo, Norway

**Keywords:** Cancer care, Content analysis, Individualized care, Nursing care plans, Nursing records, Palliative care, Person-centered care

## Abstract

**Background:**

The nursing record is essential for displaying the content and results of nursing care for persons with severe and advanced cancer in treatment and palliative cancer wards. The nursing care plan (NCP), which uses standardized terminology, organizes the nursing record. Individualization of the standards is necessary to promote person-centered care.

**Aim:**

To explore how individualized care is documented in the nursing records of persons and their families in treatment and palliative cancer care.

**Method:**

Nursing records containing NCPs and progress notes for 29 inpatients from cancer treatment and palliative wards in three hospitals in Norway were explored utilizing qualitative content analysis.

**Results:**

The NCPs elicited a limited image of the patients’ situations and care needs, mainly conveyed through standardized terminology. The progress notes appeared as the leading source of information about the patients. Three main themes emerged from the analysis: (1) unutilized opportunities for individualized documentation in the NCPs, (2) incongruence between the NCPs and the progress notes, and (3) progress notes—an alternative route for documenting individualized care.

**Conclusions:**

The study showed severe limitations in terms of the use and individualization of the NCP in the electronic health record (EHR). These limitations could be related to the cumbersome functionality of the EHR and the fact that the NCP targets efficiency and data availability purposes beyond being a tool for nursing care planning. The relational and dynamic aspects of nursing care were thinly captured, especially when documenting in a standardized format. EHR systems should be adapted to today’s technology to a greater extent and adjusted to the individual patient’s needs and experiences in cooperation with nurses as end users.

**Clinical trial number:**

Not applicable.

**Supplementary Information:**

The online version contains supplementary material available at 10.1186/s12912-025-03230-6.

## Background

The nursing record is essential to reporting the content and results of nursing care. Furthermore, the documentation serves to ensure quality during extended hospital stays. Persons with severe and advanced cancer require individualized nursing care due to the severity of the illness and the burden of the symptoms from both the illness and the treatment. An individualized approach could strengthen the patient’s well-being. The patient’s individual needs for caring and experiences of caring should be reflected in the health records from treatment and palliative cancer units [1]. Accurate documentation of the care process is critical for supporting the continuity, quality, and safety of nursing care [2, 3]. Healthcare services are shared information communities, and the electronic health record (EHR) is essential for sharing core information. The EHR is an electronic collection of medical and health information about a person, digitally stored and available for all healthcare providers taking care of the patient [4].

The nursing process, introduced by Ida Orlando in 1958 [5], is a problem-solving method for systematically assessing, planning, and evaluating the patient’s care. It was initially visualized as a handwritten nursing care plan (NCP). The introduction of electronic health journals during the 1980s and 1990s [4] enabled the development of electronic NCPs, contributing to the expansion and spread of standardized nursing terminology in nursing records. *Terminology* refers to words and terms typical of a profession, subject, or science [6]. In nursing, *standardized terminology* (ST) systems have been developed, including the NANDA International (NANDA-I) classification [7], the Nursing Intervention Classification (NIC), and the International Classification of Nursing Practice (ICNP) [8]. These systems, which represent the nursing standards in the EHR, promote consistent language and decision-making support in the documentation of nursing care [9]. However, they require individualization to the unique patient [10]. Moreover, the development of standards facilitated the analysis of nursing records as data became available for quality improvements, research, resource management, and other purposes [2]. According to Fennelly et al. [11], ST enhances nurses’ abilities to find relevant terms and improve documentation accuracy. In addition, it simplifies the possibility of reusing and tracking the nurse’s contribution to patient care and outcomes [2].

Knowledge of the person’s values, needs, resources, and experiences is essential for conducting and documenting person-centered care (PCC). PCC is high-quality care guided by the care receiver’s premises and inclusion in decision-making and care planning [12, 13]. The International Council of Nurses defines person-centered care as “valuing and respecting the characteristics, attributes and preferences of the patient, such as cultural and religious beliefs, and incorporating them into the planning and implementation of nursing care, services or program design” [14].

PCC requires the nurse’s professional competence, knowledge of the patient as a unique person, and a trusting patient-nurse relationship [15]. Documenting the partnership between the patient and the nurse can strengthen reciprocity and make the care receiver a more active partner in decision-making and care planning [12, 16]. Nursing records containing expressions of individualized care demonstrate that the care has been adapted to the person or matters vital to the person or family.

Several studies have questioned how EHR systems fit healthcare providers’ needs for functionality, workflow, and caring actions. Hardiker and colleagues [17] pointed out that the framework of EHR systems for nursing today was suboptimal and that a fundamentally new approach is needed to support nursing care planning and the patient’s journey through the hospital and treatment. Heckemann et al. [18] reported that established norms for healthcare documentation can hinder person-centered documentation. Butler et al. [19] found that contextual, goal-related, and value-based information were essential parts of clinical care; however, the clinicians expressed concerns about how this valuable information should be assessed and recorded in EHRs. A scoping review showed that many nurses found that while ST improved the accuracy of records, it was cumbersome [11]. Other studies showed that nurses found it hard to document their psychosocial caring practices in the EHR, as their knowledge, skills, and attitudes influenced their documentation practices. Limited knowledge of the patient, lack of time, and few or no options could inhibit documentation of interpersonal caring in the EHR [20, 21].

To our knowledge, few studies have explored how individualized care for inpatients in a cancer treatment or palliative unit is expressed in nursing records using NCPs. Against this background, this study aimed to explore how individualized care is documented in the nursing records of persons and their families in treatment and palliative cancer care.

## Methods

The study follows a hermeneutic approach in accordance with Gadamer [22]. A qualitative content analysis was conducted, as this approach allows for analyzing manifest (descriptive) and latent (interpretative) content [23, 24]. The Standards for Reporting Qualitative Research (SRQR) [25] were used to enhance trustworthiness.

### Data collection

Nursing records were obtained from palliative and chemotherapy inpatient cancer wards in three hospitals in Norway. Based on the inclusion criteria, the material comprised nursing records of adult inpatients diagnosed with cancer who had spent at least three days in a cancer treatment or palliative ward in the period 2015–2022. Furthermore, the records belonged to patients who had passed away.

The study requested participation from the heads of cancer departments in different hospitals in southeastern Norway, with three departments responding. The retrospective material was collected in February, April, and September 2023. The records were selected randomly according to the inclusion criteria, printed, anonymized, and copied by health personnel from the selected wards. The material was handed over to the researchers as printouts on-site. The material was marked with a code from each hospital (A, B, and C), along with the year of the data and the patients’ ages and genders. Printouts were scanned and stored in an access-controlled research server, while the paper versions were stored in a locked cabinet.

### Data material and setting

The sample contained randomly selected nursing records from 2015 to 2022 describing the hospital stays of 29 patients (*N* = 29; 12 women and 17 men). Although the number of hospital stays was limited, the material was rich, describing patients with severe or advanced cancer in a cancer treatment or a palliative context in a total of over 700 records.

**Material A** (*n* = 10) was collected from a 12-bed palliative care unit at a large university hospital, hosting patients receiving palliative and end-of-life care in the advanced phase of cancer.

**Material B** (*n* = 9) was obtained from a 28-bed unit for cancer treatment at a large university hospital. The patients had severe cancer and received chemotherapy as primary or relapse treatment.

**Material C** (*n* = 10) was collected from a 26-bed cancer unit at a medium-sized regional hospital, providing both cancer treatment and palliative care to patients suffering from severe and advanced cancer in different phases of the illness.

The patients’ ages ranged from 18 to 100 years, and the length of stay ranged from 3 to 25 days, with the mean length of stay being 9.4 days. The material included nursing records with NCPs, admission reports, progress notes, discharge reports, care plan changes, and municipal correspondence. There was a total of 157 entries and amendments to NCPs (A = 28, B = 98, C = 31) and 707 progress notes (A = 219, B = 300, C = 188) covering information from day, afternoon, and night shifts in a free-text format.

The nursing records were provided in the EHR system DIPS (Distributed Information and Patient Data System in hospital, authors’ translation) [26]. The records were organized based on NCPs, including nursing diagnoses (NANDA-I), outcomes, and interventions (NIC). The NCP evaluations were represented in the progress notes in a free-text format. Knowledge-based standardized care plans were available in the EHRs as a clinical decision support system. The nursing diagnoses, outcomes, and interventions in the NCPs and the texts of the progress notes were categorized based on numbered functional areas [27].

### Data analysis

The content analysis was conducted in accordance with Graneheim and Lundman [23]. The material’s scope was organized into an Excel spreadsheet to make an overview, followed by a thorough and repeated reading of the material on paper. Handwritten memos followed the reading, and as the analyzing process developed, text passages were marked as meaning units – parts referring to equivalent content – and coded using different colors. All meaning units were manually outlined in a new Excel spreadsheet. The nature of our data material—the different texts written by the nurses in the nursing records—already appeared as a condensed and, to some extent, abstracted text [24] and was kept as meaning units. We wanted to keep the formulations as close to the text as possible to retain the sense of the data in our further analysis. Therefore, the meaning units were first coded and then sorted into categories according to manifest content and low abstraction, as described by Graneheim, Lindgren, and Lundman [24, 28]. The first author has a clinical background as a cancer nurse and is currently a Ph.D. student. The second and third authors are both professors in nursing with long experience in nursing and health science research. Two of the authors are also familiar with nursing records from clinical practice. All the authors read parts of the material, and through repeated discussion among the authors and by working between the whole and the parts of the text material, the main themes emerged as abstracted and latent content of the material [28]. The analytical process is illustrated in Table 1.

**Table 1:** Examples of the analytical process

Meaning units	Subcategories	Categories	Subthemes	Themes
The patient says that he has had increasing anxiety and panic. Yesterday, he had the feeling that he was drowning and that he was not going to survive this. He says he feels much better now that the pleural vein was inserted.	Statement from the patient as a quote	The patient’s voice	Natural language conveying the voice of the patient	Progress notes—an alternative route for documenting individualized care
His wife and three sons have been to a family information consultation with the physician. It is crucial for all of them to be present when the patient dies. It takes the wife approx. one hour to get here, so it is important to notify early in the case of a change.	Presence with the beloved person	Matters of importance for the family		
He had been out of bed in his own clothes and had a training session. He plans the day with nutrition/activity/rest in mind.	Maintain routines and normality	Matters of importance for the patient	Glimpses of the person	

### Ethical considerations and approvals

Restrictive procedures regulate the use of research data such as nursing records. Our concern was protecting the anonymity of the patients and healthcare providers in the material. The Norwegian Regional Ethical Committee (REK) approved the study (ref. no. 504500) but allowed only the collection of nursing records from patients who were deceased at the time of retrieval. Permission to retrieve the records was sought from the Norwegian Agency for Shared Services in Education and Research (SIKT in Norway; ref. no. 612395), the cancer department heads, and the local data protection officers at each hospital. All identifying information about the patients, relatives, and healthcare providers were anonymized.

## Results

The following themes emerged through the analysis: (1) unutilized opportunities for individualized documentation in the nursing care plan, (2) incongruence between the nursing care plan and the progress notes, and (3) progress notes—an alternative route for documenting individualized care. Corresponding subthemes elaborated the content of the themes (see Table 2).

**Table 2:** Themes and subthemes

	Themes	Subthemes
1.	Unutilized opportunities for individualized documentation in the nursing care plan	The dominance of treatment-centered information in the nursing care plan
		Lack of the patient’s perspective in the individualization of the nursing care plan
2.	Incongruence between the nursing care plan and the progress notes	
3.	Progress notes—an alternative route for documenting individualized care	Natural language conveying the voice of the patient
		Glimpses of the person

### Unutilized opportunities for individualized Documentation in the nursing care plan

The analysis of the NCPs revealed a limited presentation of the patients’ situation and care needs. However, this varied in content and comprehensiveness between the three wards and in the frequency of the entries and changes. Ward A (palliative) used NCPs with ST, but only to some extent, and the NCPs mainly conveyed information about medical treatments and technical equipment. The excerpts from Ward B (cancer treatment) showed more frequent use of NCPs with ST. Many patients in this sample had previous unit admissions and existing NCPs, which were updated to reflect the recent treatments upon new admission. However, updates of the NCPs were random during hospital stays. Ward C (general cancer) used NCPs to a lesser extent, and ST appeared to be seldom and randomly used. Some NCP entries were written in a natural language, not with ST, and some information was written as uncategorized messages in an open field in the NCP. Two subthemes served to elaborate this theme’s content.

### The dominance of treatment-centered information in the nursing care plan

A general trend was that the NCPs’ elements were mainly adapted to tasks related to patients’ expected treatment needs, the side effects, and the burden of advanced cancer symptoms. An illustration could be the palliative Ward A’s frequent use of *NANDA 00133: chronic pain*, followed by the outcome formulations *pain relieved in rest/pain relieved by activity* and *NIC 2300: medication administration—use of Graceby pump (specify).* In several excerpts, this diagnosis–outcome–intervention triangle was listed with no individualizing information, giving a task-like impression, reinforced by the fact that the only measures seemed to be the administration form information.

**Fig. 1:**
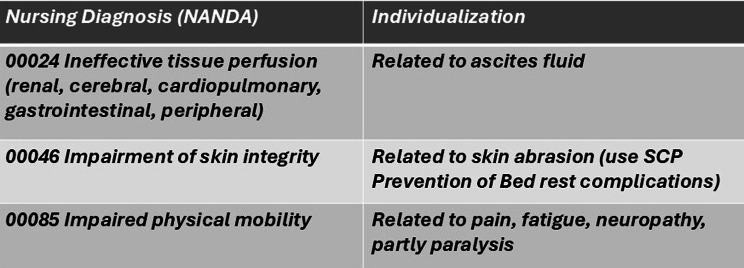
Part of an NCP from Excerpt 6 C, Afternoon Shift—Individualized Nursing Diagnosis

The individualization of ST was sparse, though varying between the three wards. The analysis showed that the more the nurse individualized the standards, the more precise the image of the patient’s functionality and situation that the text elicited. The adaption of nursing care mostly appeared by adding information to a nursing diagnosis (NANDA), as in the following case (see Fig. 1):

The NANDAs in this NCP contributed to the individualization of the nursing diagnosis by adding descriptions of the patient’s capabilities and health condition. Through this adaption, the nurse shared knowledge of the patient that was necessary for providing person-centered care.

**Fig. 2:**
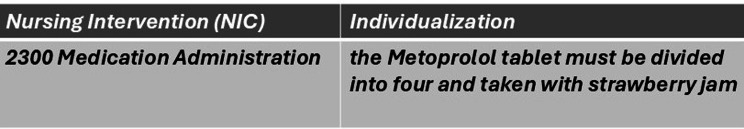
Excerpt 20B, Night Shift—Individualization of NIC

**Fig. 3:**
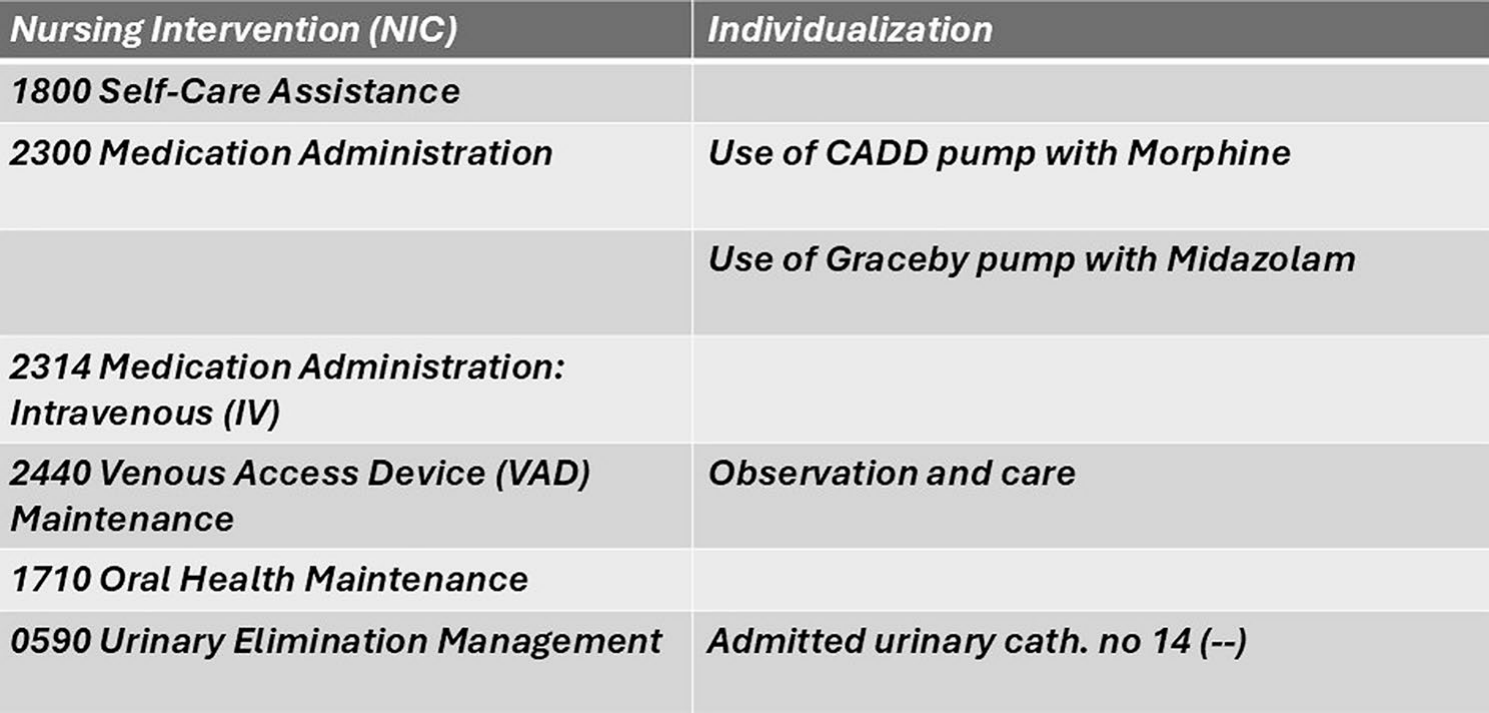
Excerpt 4 A, Afternoon Shift—Documenting Medical Devices

Nursing interventions (NICs) often followed the nursing diagnoses in the excerpts, as if responding to the needs displayed by the NANDAs. Several NCPs had NICs without this informative nursing diagnosis. Single NICs were sometimes formulated as a message or requested action. The interventions were mainly described using standardized terms; however, a few times, individualization was added with descriptions of the patient’s wishes and care needs as well as practical information, as in the following case (see Fig. 2):

Another workflow-supportive example of the individualization of NICs was the documentation of the medical or technical equipment in use for the patient: the kind of connected continuous infusion pump, the size of the urinary catheter, and the type of central venous access (CVA). In the following example (see Fig. 3), representing a young male palliative patient, the type of infusion pump was documented, along with other measures:

The task focus of the NCP became stronger when only actions relating to biomedical and physiological health were documented, leaving an impression of the patient’s body as just an object in need of physical nursing actions. Subjective information about the young man surrounded by his family, living the last days of his life, was missing from the NCP.

### Lack of the patient’s perspective in the individualization of the nursing care plan

**Fig. 4:**
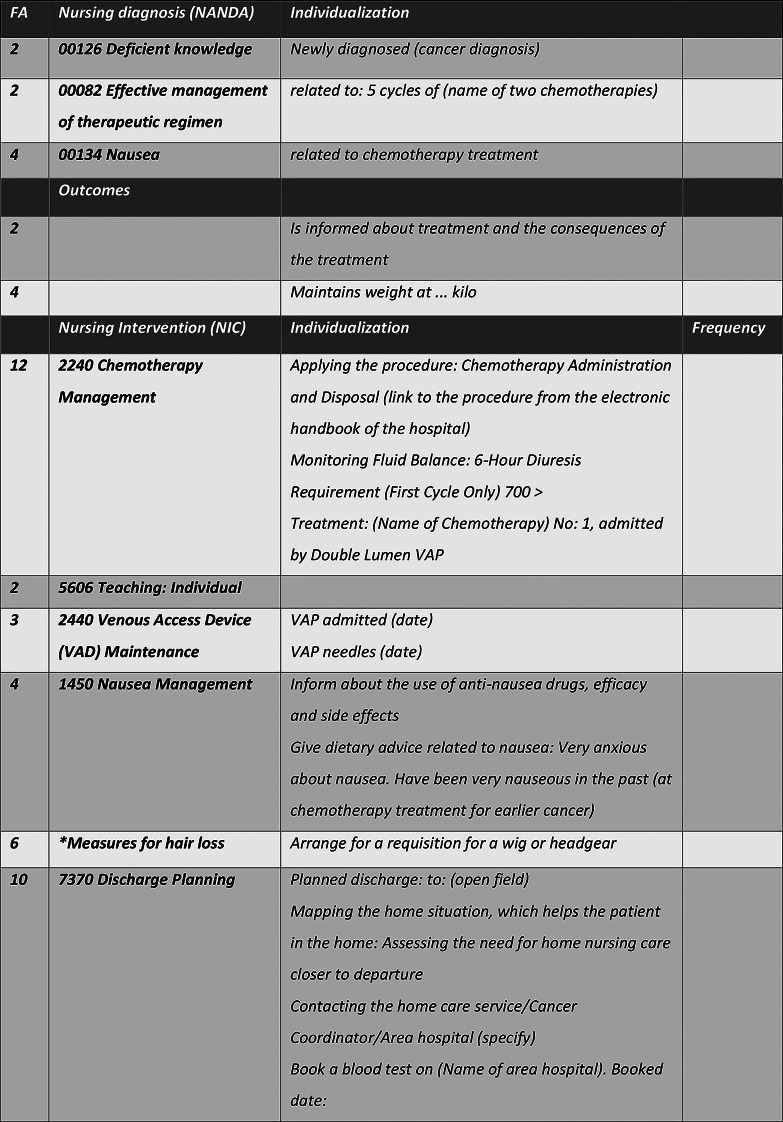
Excerpt 15B, Admission Note and Following Afternoon Shift—Individualization of Standardized Care Plan

When NCPs were initiated, most entries included equivalent codes and terms for all patients in the same situations (i.e., chemotherapy treatment or advanced palliative care). The wards’ standardized care plans seemed to be a source of maintaining the NCPs based on patients’ excepted diagnoses and care needs. The following illustration (see Fig. 4) involved a comprehensive NCP created on the day when a female patient was admitted to her first chemotherapy treatment cycle. According to the admission notes, the woman had previously been through another type of cancer and had been struggling with the chemotherapy treatment. The day shift nurse initiated the NCP, and the afternoon nurse developed it further.

This NCP offered information about the treatment, specified in terms of the patient’s diagnosis and the whole treatment plan; furthermore, information and specific observations related to the current treatment and venous access were added to the plan. *NIC 2240: chemotherapy management* was connected to *NANDA 00082: effective management of the therapeutic regimen*, specifying nursing actions regarding the chemotherapy treatment. *NIC 1450: nausea management* was connected to *NANDA 00134: nausea* and individualized through information regarding the patient’s experiences and worries about repeating symptoms, and this passage informed readers of the NCP to pay attention to this. *NIC 7370: discharge planning* focuses on the home situation after the first treatment cycle, considering contact with municipal home care, local hospitals, and other municipal-based healthcare providers. The NCP expressed a planning attitude, with a long-term perspective of care for the woman throughout the treatment. The plan was adapted to this woman’s nursing care needs—but it did not reflect any information on what mattered for her concerning emotional, psychosocial, or spiritual matters, even though she had been newly diagnosed with cancer for the second time. Notably, we found the exact same formulas in the records of other patients in similar situations, including in terms of the NANDAs and NICs and some of the individualizing texts.

This NCP mainly demonstrated the use of standardized care plans as a tool for documenting nursing. This decision support tool could hardly be used as a guide for PCC, as it, in this case, focused on the treatment and side effects, leaving out other aspects of importance for the person involved. The information in *NIC 1450: nausea management* was an exception in this regard, as the text offered individualized information concerning the woman’s anxiety about nausea related to her earlier experience of cancer treatment.

### Incongruence between the nursing care plan and the progress notes

The analysis of the nursing records showed that when entries or changes were carried out in the NCPs, the same actions were often described in the progress notes from the same day. Thus, the NCPs functioned as real-time documentation; however, some overlapping or redundancy was found between the NCPs and the progress notes. In some cases, the NCP was initiated or changed several days after the progress notes described changes in the patient’s condition. Hence, these entries in the NCP could be interpreted as less relevant.

The progress notes also revealed several changes that were not followed by NCP entries. In the case of a female patient from Ward A (palliative), the progress notes stated that the woman was a Muslim, came from another culture, and hardly spoke Norwegian, adding that the family wanted a religious follow-up. The woman was surrounded by her family all day and had considerable pain and many symptoms. Her cancer gradually deteriorated throughout her 11-day stay, but there were no updates in the NCP despite the seriousness of the changes in her condition. In another case involving a male patient from Ward B (treatment), a comprehensive NCP was made on the day that he started the isolation phase after high-dose chemotherapy with stem cell support, but no changes were made to the NCP until discharge two weeks later.

The progress notes for both patients contained multiple changes in their conditions and several recommended actions. This incongruence could undermine the NCPs’ trustworthiness and create uncertainty about the patients’ situations, as NCPs are expected to be the primary source of information on patients’ conditions and the fastest way to orient oneself at the start of a new shift. The incongruence between the NCPs and progress notes also represented a risk to patient safety.

### Progress notes—An alternative route for documenting individualized care

The progress notes contained the nurses’ actions and statements regarding the patients and distributed messages for the next shift, thereby ensuring patient care continuity. Information from other healthcare providers was sometimes repeated in the progress notes. Through the analysis of the nursing records, the progress notes stood out as the leading way of documenting nursing care. The progress notes were written in a natural language and a free-text format, systematized only according to the numerical functional areas described by DIPS. As the progress notes often contained information not reported in the NCP, they appeared less as an evaluation of the NCP. Thus, the NCP and the progress notes seemed to be two independent parts of the nursing record, without functioning as an integrated whole. In some cases, the patient’s quotes, wishes, and statements about what was important to them were documented in the progress notes. These elements appeared as text fragments, expressing the patient’s perspective and the patient-nurse relationship as essential aspects of the care.

### Natural Language conveying the voice of the patient

In the progress notes, nurses described patients’ verbal and non-verbal expressions. Many statements from patients were documented as quotes, as shown in the following case from Ward A (palliative):

*The patient says he has been tired today. He says he feels more lethargic today and does not want to feel so tired and lethargic; he wants to be more involved. The patient thought there was an increase in morphine and midazolam today and wanted to decrease it. I explained that there was increased morphine yesterday and midazolam during the day. The patient explained that he was not fully present during the ward round and had not received all the information.* (6 A, day shift)

This record contained a dialogue between the patient and the nurse, and the patient’s experience was the fundament of the record, made possible by the free-text format. The nurse documented how she guided the patient and explained the recent medicalization. Nearly all the sentences started with the phrase *“The patient says*,*”* and the patient’s voice gives the reader the impression that the nurse acknowledged and wanted to share the patient’s experience with other healthcare providers taking care of the patient.

The use of the patient’s and family members’ names in the progress notes contributed to a personal approach in the documentation, as in this excerpt from Ward A (palliative): *“I moistened her mouth with lemon water and ice*,* and [name] felt it was pleasant*” (5 A, evening shift). In this case, the patient appeared as a person for the text’s reader, as the person’s name replaced the *patient* concept. By writing the personal pronoun “I” and the patient’s name, the nurse gave the impression of a close patient-nurse relationship, which could be displayed in the NCP only to a lesser degree. The relationship was confirmed by describing the patient’s reaction as “pleasant,” including the patient’s subjective experience of the nurse’s action.

### Glimpses of the person

In some cases, the nurses’ texts in the progress notes contained descriptions of the patients’ lives outside the hospital, such as their previous work, their home situations, and the things that were meaningful to them. It is reasonable to anticipate that the information emerged either because the nurses had asked how the patients were doing or because the patients had initiated dialogues about spiritual or mental concerns, thoughts, or feelings. Thus, the descriptions elicited glimpses of the persons in their natural environments, making visible the vulnerable human beings in a life crisis.

This kind of individualized information about patients did not appear in the NCPs in the sample. In one record, in which a patient undergoing a long-term chemotherapy treatment returned from a short leave from the hospital, the nurse wrote as follows in the progress notes: *“The patient has had good days at home*,* has been in good shape*,* and has done some gardening”* (18B, afternoon shift). As the nurse mentioned gardening, she conveyed information relevant to the patient as a whole person in his social context at home, not just as a hospitalized patient with cancer. The description of the gardening could be interpreted as essential to the patient’s well-being, interests, and experience of responsibility for his property. At the same time, it revealed that the nurse considered this information essential to caring for the person involved.

Another progress note described a male palliative patient with severe fatigue and pain, providing the reader with a description of something that mattered to the patient—keeping his dignity and handling his intimate zones by himself:

*Peed in the bathroom once. Concentrated urine. I remember the patient being a little “shy” from a previous admission and saying he wanted to arrange the most private things himself. When I asked him about a urinary catheter today*,* he replied*,* “No*,* it hasn’t come that far.” However*,* I see he uses much strength when he goes to the bathroom*,* and I am thinking it might be appropriate for a male nurse to handle the problem with a catheter and that it is a man who inserts it? Only a suggestion. I got the patient to agree that the nurse [male colleague*,* anonymized name] had added a urisheath. He confirmed that he wanted a man to do it.* (2 A, evening shift)

The patient cited above rejected the offer of a urinary catheter, insisting that he was not so sick that he needed this equipment. The nurse described how she did not enter his most private zones, suggesting a male colleague help the patient instead. The nurse’s description of her actions to prioritize the patient’s strength and at the same time attend to his dignity and respect was an example of documentation of individualized care that supported the patient’s values.

## Discussion

This study explored how individualized care was documented in the nursing records for persons and families in cancer care. Here, we discuss how the functionality of the EHR can influence the recording process and documentation of individualized care in a cancer treatment or palliative care setting where patients suffer from compound and comprehensive symptoms. Furthermore, we consider if the nature of caring in nursing in cancer care is too complex to capture through standardized texts in nursing records.

### Functionality of the EHR and the nursing record process

Our study showed that the nurses utilized the NCPs to a limited extent. Individual adaptations of the standard texts were few and rarely comprehensive, and the NCPs were not systematically updated throughout the patient’s hospital stay. According to the guidelines for nursing documentation, the structured format should provide the main document of the nursing record, and the NCP should be person-centered and reflect the person’s needs by describing individual variation and evaluation of care [16]. However, using standardized terms alone can lead to misinterpretations of the patient’s nursing care needs [10]. The possibility of adding individualizing characteristics to nursing diagnoses and interventions was included in the current software, and the NCP, supported by standardized care plans, offers several options for documenting the patient care with great accuracy [26]. Ideally, an EHR system should allow for data reuse, facilitate knowledge-based practice, and provide an overview of the need for or provision of healthcare [2]. Health authorities and international nursing organizations agree on the need for standardization in nursing informatics [8, 16], as this can improve the quality of clinical processes, facilitate the workflow, and support the exchange of information between nurses to promote care continuity and coordination [2].

By contrast, our data analysis revealed that the nurses circumvented the barriers of the standardizations and the NCP through their complementary narratives in the progress notes. Compliance with the system was handled by documenting nursing care in free text. These findings indicate that the nurses’ loyalty lay in documenting what they considered essential for the patient’s situation, even if this did not align with the EHR guidance. We could raise the question of how well the strictly structured NCP functions in practice. Furthermore, the task-oriented EHR seems more limiting than inviting for documenting the core of nursing [29, 30], which is dynamic, complex, and grounded in individualized care for the person in need [31, 32]. Moreover, Butler et al. [33] argued that EHRs are hardly designed to document patients’ goals or other person-centered information as structured data. They found that nurses individualized the care in progress notes by citing the patients, supporting their goal process, and documenting matters of importance in their social context, using natural language. Additionally, the study of Hardiker et al. [17] pointed out that EHRs barely support the needs of clinical nursing practice, nor do EHRs facilitate nurses’ workflow.

Our study indicates that the effectiveness of electronic documentation seems unrealized. The records in our study were mainly conducted in the traditional way by using natural language in a free-text format in the progress notes. The findings also reveal that the texts summarized the nurses’ actions more than describing patients’ perspectives. Using the NCP with standards requires extended knowledge of nursing informatics [34, 35]. Previous studies showed that nurses found it time-consuming and demanding to individualize ST [36], experiencing the standards as cumbersome [11]. Utilizing the EHR system’s functionality required calm and concentrated time to analyze, reflect, and plan the care [37]. This could be a challenge due to the workload of a palliative or chemotherapy unit. The unit managers are responsible for providing an environment that facilitates nursing care planning. Nursing documentation could be seen as an expression of professional nursing practice, and by clarifying and facilitating appropriate routines, the unit managers could help prioritize individualized documentation through their expectations, training, and colleague support.

According to the study of Castellà-Creus et al. [37], the barriers to individualizing the standardized care plan included an excessive workload, the complexity of care, and the limited involvement of nursing managers. Insufficient knowledge of using the standardized care plan, a lack of interest, and low motivation led to free-text recording rather than adding and changing elements of the NCP [37]. Our material does not offer an answer concerning nurses’ competencies or motivation. However, we agree with the argument that if nurses are not confident with the NCP format, necessary measures to increase nurses’ proficiency in documenting in a standardized format should be initiated, such as follow-up, guidance, and ward-based education [35]. Lydahl et al. [38] described such an initiative, showing how a person-centered training program enhanced nurses’ abilities to document individualized goals.

### Individualized caring in nursing seems complex to capture by text

The findings imply that natural language facilitated documentation of the patient’s perspective in the nursing records and sometimes made the relationship between the patient and the nurse visible. These findings are similar to those reported by Laitinen et al. [39], who identified the three aspects of the patient’s perspective in the progress notes: the patient’s voice, the nurse’s view, and statements of a mutual view in the patient-nurse relationship. Reporting patients’ voices may strengthen their possibilities for participation in care, which is fundamental for strengthening their empowerment, health, and well-being. The value of the patient’s perspective is based on an understanding of nursing as caring that involves a relationship between the nurse and the patient, embracing the patient as a unique person [31]. The rare traces of this relationship in the nursing records may be related to caring as an experience between the patient and the nurse [31], an experience that seems hard to express by text. The structured records focus on biomedical problems and seldom include dialogical and narrative expressions of the patient’s experiences [29]. Standardized texts can hardly capture the recording of caring in nursing, as natural language is the language of the heart, carrying the tone of the meeting required to describe relational dimensions of nursing care [32]. Moreover, De Groot et al. [40] recommended that there should always be possibilities for narrative texts detailing the individual patient’s unique situation.

Caring involves a meeting between the patient and the nurse as persons [32]. Patients need to feel confident to share their stories [32], and a trusting relationship is fundamental in patient-nurse interactions. Studies have shown that the nurse’s competencies contribute to the patient’s feeling of security, helping the patient perceive the relationship as caring [15]. Suppose the content of the nursing record solely focuses on the patient’s medical conditions and the tasks in a standardized format. In that case, it may hinder nurses from prioritizing and documenting other nursing actions, such as assessment of the patient’s capacities for participation and shared decision-making. The biomedical focus objectifies the patient as a physical body with cancer and could thereby potentially impair the nurse’s consciousness of the fundament of nursing: the caring relationship between the patient and the nurse [32]. Based on the authors’ clinical experience, we suggest that the lack of a culture among nurses for describing caring encounters could also explain the limited recording of PCC.

Our study reveals a uniform picture of the material, which contained limited documentation of psychosocial, emotional, and spiritual care in the NCPs but with more complementary formulations in the progress notes. However, according to the studies of Hynnekleiv et al., details of the patients’ concerns remained scarce even in the records of the dialogues between patients and nurses [41]. Lydahl et al. [38] found that the primary purpose of nursing records seemed to be safeguarding the communication between different healthcare providers rather than illuminating the patient’s values and experiences or the communication between the patient and healthcare providers. Thus, individualized documentation implies a fundamental challenge to the healthcare providers’ focus, as this relational, concrete, and often tacit side of nursing care can be hard to capture in words [19, 42]. Long-term care studies have demonstrated this challenge, showing that nursing diagnoses were often documented in short statements about the residents’ conditions in a free-text format [36] and that the records lacked detailed individualization in the initial assessments and care planning [43].

We argue that the NCP should be set up by a nurse who knows the patient to secure the individualization of the NCP and meet efficiency and quality requirements. Another potential way of presenting the patient’s individuality is to allow them to report their values, needs, and experiences in the EHR themselves, as in the innovative self-reporting scheme “This Is ME” by Sequeira et al. [44]. By facilitating the patient’s narrative in the EHR, the scheme increased the person-centeredness of the EHR, as the patient’s perspective was directly present in the records.

There is a need to strengthen nurses’ competencies in formulating individualized nursing care by recording and putting the patient’s perspective into words. To attain this goal, these competencies need to be highlighted both in the nursing curriculum and in nursing practice [34]. Shared stories of the lived experience of nursing practice are well suited to strengthening one’s awareness of oneself as caring, as the articulation and sharing of stories from nursing practice enhance reflection and create ideas on how to document essential aspects of caring in nursing records [31].

### Strengths and limitations

The SRQR checklist for qualitative research was applied to enhance trustworthiness [25]. The analysis was conducted by all three authors, working back and forth between the parts and the whole of the material to secure dependability. The first and final authors’ familiarity with nursing records from clinical practice may have improved the study’s trustworthiness. Transferability was facilitated by an accurate and rich description of the context of the data and the recent EHR system, as well as by the fact that the material contained both NCPs and progress notes [28]. By including nursing records from both treatment and palliative care wards, the study gained access to rich data, as records from both women and men in different life situations and at various ages were explored. The nurses’ original expressions were used as meaning units and quotes. Together, this enhanced the study’s credibility [24, 28].

The scope of the study was one of the study’s limitations, as the material only covered nursing records from 29 patients. Not all shifts had records, and the length of some of the patients’ hospitalizations was thus estimated by perusing the type of shift. Another limitation was that all the material represented the same EHR system, which could reduce the transferability of the results, as other EHRs might have organized the nursing records differently and presented other kinds of usability. The focus on the content and the structure of the nursing records contributed to reduced attention to the documentation process, which was not investigated. Finally, the nursing records carried a limited picture of reality and contained solely what the nurses considered essential to be written in the EHRs, which was the fundament of this study.

## Conclusion

This study explored how individualized care was documented in the nursing records in the EHRs of persons and their families in cancer care. Three main themes were identified as essential aspects of the nursing records: (1) unutilized opportunities for individualized documentation in the nursing care plan, (2) incongruence between the nursing care plan and the progress notes, and (3) progress notes—an alternative route for documenting individualized care. The study identified severe limitations in the general use of the NCPs and the specific possibilities for documenting psychosocial, existential, and relational aspects of nursing in them. These limitations should be paid more attention to in the further development of nursing informatics.

The standardization of electronic nursing records enables the analysis of the nurse’s contribution by making data available for quality improvements, research, and resource management [2]. In this way, the NCP targets purposes beyond being a tool for planning nursing care for the individual patient. Through the NCP’s support for the benefits of standardization, the capacity to record individual nursing may be impaired, as an all-standardized format can hardly capture the dynamic caring of nursing.

### Implications for practice

The results of this study challenge nurses to develop NCPs further by adapting ST to display their individualized care. Documentation using NCPs and ST requires extended knowledge of nursing informatics. As preparation for the documentation of individualized care, nursing informatics and training in personalized adaptation of ST should already be included in the nursing curriculum [34]. Nurses also need to be involved in technology development, as their end-user experience and clinical competence make them capable of identifying risks with technology and scientific advancements and implementations [14, 17].

EHR systems should be adapted to today’s technology to a greater extent and adjusted to the individual patient’s needs and experiences in cooperation with nurses as end users. Possibilities for free text in the nursing record should be continued, as essential parts of caring in nursing are hard to formulate through standards. Nursing managers should allow time and training on using the NCP and individualizing documentation, as well as facilitating ward-based reflection on essential aspects of nursing practice and how to document these in the NCP.

When considering the next research steps, eliciting cancer and palliative nurses’ perspectives of nursing and the need for nursing documentation in treatment and palliative settings might be relevant. Similarly, nursing management’s perspective might illuminate and clarify the challenges of nursing recording in cancer care.

## Electronic supplementary material

Below is the link to the electronic supplementary material.

Supplementary Material 1

## Data Availability

The data sets used and analyzed in this study are unavailable due to the protection of the study participants’ privacy following the project approval by the Regional Committee for Medical Research Ethics in Norway.

## Abbreviations

CVA: Central venous access
DIPS: Distributed Information and Patient Data System in Hospital (authors’ translation). In Norwegian: Distribuert Informasjons og Pasientdatasystem i Sykehus
EHR: Electronic health record
ICNP: International Classification of Nursing Practice
NANDA-I: NANDA International, Inc. is a brand name. North American Nursing Diagnosis Association was the name before 2002
NIC: Nursing Intervention Classification
NCP: Nursing care plan
PCC: Person-centered care
SCP: Standardized care plan
ST: Standardized terminology
VAP: Venous access portal (Port-a-Cath^®^)
